# E-NPP3 controls plasmacytoid dendritic cell numbers in the small intestine

**DOI:** 10.1371/journal.pone.0172509

**Published:** 2017-02-22

**Authors:** Yoki Furuta, Shih-Han Tsai, Makoto Kinoshita, Kosuke Fujimoto, Ryu Okumura, Eiji Umemoto, Yosuke Kurashima, Hiroshi Kiyono, Hisako Kayama, Kiyoshi Takeda

**Affiliations:** 1 Laboratory of Immune Regulation, Department of Microbiology and Immunology, Graduate School of Medicine, WPI Immunology Frontier Research Center, Osaka University, Suita, Osaka, Japan; 2 Core Research for Evolutional Science and Technology, Japan Agency for Medical Research and Development, Tokyo, Japan; 3 Department of Gastroenterology and Hepatology, Graduate School of Medical Sciences, Kumamoto University, Kumamoto, Japan; 4 Division of Mucosal Immunology, Department of Microbiology and Immunology, Institute of Medical Science, The University of Tokyo, Tokyo, Japan; Keio Gijuku Daigaku, JAPAN

## Abstract

Extracellular adenosine 5’-triphosphate (ATP) performs multiple functions including activation and induction of apoptosis of many cell types. The ATP-hydrolyzing ectoenzyme ecto-nucleotide pyrophosphatase/phosphodiesterase 3 (E-NPP3) regulates ATP-dependent chronic allergic responses by mast cells and basophils. However, E-NPP3 is also highly expressed on epithelial cells of the small intestine. In this study, we showed that E-NPP3 controls plasmacytoid dendritic cell (pDC) numbers in the intestine through regulation of intestinal extracellular ATP. In *Enpp3*^-/-^ mice, ATP concentrations were increased in the intestinal lumen. pDC numbers were remarkably decreased in the small intestinal lamina propria and Peyer’s patches. Intestinal pDCs of *Enpp3*^-/-^ mice showed enhanced cell death as characterized by increases in annexin V binding and expression of cleaved caspase-3. pDCs were highly sensitive to ATP-induced cell death compared with conventional DCs. ATP-induced cell death was abrogated in *P2rx7*^-/-^ pDCs. Accordingly, the number of intestinal pDCs was restored in *Enpp3*^-/-^ *P2rx7*^-/-^ mice. These findings demonstrate that E-NPP3 regulates ATP concentration and thereby prevents the decrease of pDCs in the small intestine.

## Introduction

Extracellular ATP released by damaged/dying or activated cells regulates several immune responses via purinergic receptors such as P2X and P2Y [1–3]. ATP activates many types of immune cells, including dendritic cells (DCs), macrophages, T cells, B cells, mast cells, and basophils, via P2Y and P2X receptors [4–10]. Furthermore, several lines of evidence have indicated that extracellular ATP induces inflammatory responses in several tissues [11–14]. In addition, ATP induces apoptosis of several cell types [15–21]. Thus, regulation of the extracellular ATP concentration is required to maintain host homeostasis.

Nucleotide converting ectoenzymes, such as ecto-nucleoside triphosphate diphosphohydrolase (E-NTPD) and ecto-nucleotide pyrophosphatase/phosphodiesterase (E-NPP) families, play critical roles in controlling the concentration of extracellular ATP through ATP hydrolysis [22–27]. Additionally, several studies have indicated that nucleotide converting ectoenzymes modulate immune responses. E-NTPD1 and ecto-5'-nucleotidase (CD73) are highly expressed on CD4^+^ Foxp3^+^ regulatory T (Treg) cells, and cooperation of E-NTPD1 and CD73 enhances the immunosuppressive activity of Treg cells via the generation of adenosine from extracellular ATP [28,29]. Moreover, E-NTPD1 and CD73 assist B cells to enter the class switch recombination process by converting ATP to adenosine [30]. E-NTPD7 is specifically expressed in epithelial cells of the small intestine, and inhibits ATP-mediated Th17 cell responses [31]. In addition to E-NTPD1, CD73, and E-NTPD7, E-NPP3 was recently shown to modulate immune responses. E-NPP3, which is expressed on activated basophils and mast cells, suppresses ATP-induced activation of basophils and mast cells, and prevents chronic allergic inflammation [10]. Thus, the functions of E-NPP3 have been well established in basophils and mast cells. However, it remains unclear which functions are performed by E-NPP3 expressed on other cell-types.

Plasmacytoid dendritic cells (pDCs), which express CD11c at a low level but highly express PDCA-1 and B220, play an essential role in the host defense against viral infection by secreting large amounts of type I interferon (IFN) and IL-12 [32,33]. pDCs are largely distributed to secondary lymphoid tissues such as lymph nodes and the spleen. However, they are also present in lamina propria of the small intestine, where they express the gut homing receptor C-C chemokine receptor (CCR) 9 [34]. CCR9 expression correlates with the tolerogenic property of pDCs [35]. pDCs in gut-associated lymphoid tissues, such as Peyer’s patch (PP) and mesenteric lymph nodes (MLNs), possess unique properties. These properties include a low level of type I IFN production, suppression of intestinal inflammation and induction of IgA class switching of B cells [36–38]. However, the regulation of intestinal pDC activity remains unclear.

In this study, we investigated the immunomodulatory functions of E-NPP3 in the intestine where E-NPP3 was highly expressed. Mice lacking E-NPP3 showed an elevated concentration of luminal ATP in the small intestine and a decrease in pDC numbers specifically in PPs and lamina propria of the small intestine. pDCs were highly sensitive to ATP-induced cell death that was dependent on P2X7. Accordingly, introduction of *P2rx7* deficiency in *Enpp3*^*-/-*^ mice restored the pDC number in the intestine. These findings demonstrate that E-NPP3, which is highly expressed in epithelial cells of the small intestine, plays a critical role in the maintenance of pDC cell numbers through hydrolysis of luminal ATP.

## Materials and methods

### Mice

The protocols used for all animal experiments in this study were approved by the Animal Research Committee of Osaka University, Japan (No. 23-076-01). *Enpp3*^*-/-*^ and *P2rx7*^*-/-*^ mice were generated as described previously [10,13]. *Kit*^*W-sh/W-sh*^ mice were kindly provided by Dr. H. Suto (Atopy Research Center, Juntendo University, Japan). These mice were backcrossed to BALB/c for at least seven generations. BALB/c mice were purchased from Japan SLC (Shizuoka, Japan). Mutant mice and their wild-type littermates at 8–12 weeks of age were used in experiments. These mice were maintained under specific pathogen-free conditions. For some experiments, germ-free BALB/c mice were purchased from Clea (Tokyo, Japan).

### Reagents

An annexin V staining kit, active caspase-3 staining kit, anti-mouse PerCP/Cy5.5-CD45RA (14.8) and CD16/32 (2.4.G2) antiboies were purchased from BD Biosciences (San Diego, CA, USA). Anti-mouse PE-CD45 (30-F11), Pacific Blue-CD45 (30-F11), PerCP/Cy5.5-CD4 (GK1.5), FITC-CD4 (GK1.5), FITC-CD8a (53–6.7), APC/Cy7-CD45R/B220 (RA3-6B2), FITC-CD45R/B220 (RA3-6B2), Alexa Fluor 647-CD317 (129C1), PE/Cy7-CD11c (N418), FITC-CD11c (N418), APC-FcεRI (MAR-1), PE/Cy7-CD117 (2B8) and PE-Siglec H (551) antibodies were purchased from Biolegend (San Diego, CA, USA). Anti-mouse FITC-CCR9 (eBioCw-1.2) antibody was purchased from eBioscience (San Diego, CA, USA). Anti-mouse FITC-CD3e (145-2C11) antibody was purchased from Tonbo biosciences (San Diego, CA, USA).

### Quantitative RT- PCR

Total RNA was isolated using TRIzol reagent (Sigma, St Louis, MO, USA), and reverse transcribed with Moloney murine leukemia virus reverse transcriptase (Promega, Madison, WI, USA) and random primers (Toyobo, Tokyo, Japan) after treatment with RQ1 DNase I (Promega). Quantitative real–time PCR was performed using Go Taq qPCR Master Mix (Promega) in a Step One Plus (Applied Biosystems). Amplification conditions were: 94°C (5 min), followed by 40 cycles of 94°C (20 s), 55°C (20 s), and 72°C (50 s). All values were normalized to the expression level of *Gapdh*. The following primer sets were used: *Enpp3*: 5’-CTCATGCCCTGCACTACAGA-3’ and 5’-TAGCCTTTGGTTTGCTTGCT-3’, *Gapdh*: 5’-CCTCGTCCCGTAGACAAAATG-3’ and 5’-TCTCCACTTTGCCACTGCAA-3’, *P2rx1*: 5′-ACGAAACAAGAAGGTGGGAGT-3′ and 5′-AGGCCACTTGAGGTCTGGTAT-3′; *P2rx2*: 5′-GAGAGCTCCATCATCACCAAA-3′ and 5′-CAGGGTCTGGGAAGGAGTAAC-3′; *P2rx3*: 5′-CCGAGAACTTCACCATTTTCA-3′ and 5′-TTTATGTCCTTGTCGGTGAGG-3′; *P2rx4*: 5′-TGGCTACAATTTCAGGTTTGC-3′ and 5′-GATCATGGTTGGGATGATGTC-3′; *P2rx5*: 5′-AACCGTCTGGACAACAAACAC-3′ and 5′-TTTCATCAGGTCACGGAACTC-3′; *P2rx6*: 5′-CTCCTGGAGGTGGTTCATGTG -3′, 5’-GGCTTTGGCAAGCTTTACTTC-3’; *P2rx7*: 5’-TGTGTGCATTGACTTGCTCA-3’ and 5’-CTTGCAGACTTTTCCCAAGC-3’; *Ifna1*: 5’-TGGTCCTGGCGGTGATGAGC-3’ and 5’-AGGGATGGCTTGAGCCTTC-3’; *Il12b*: 5’-GGTTTGCCATCGTTTTGCTGG-3’ and 5’-CATCTTCTTCAGGCGTGTCAC-3’.

### Generation of a monoclonal anti-mouse E-NPP3 antibody

Mouse E-NPP3-expressing NIH 3T3 cells were injected into the footpads of *Enpp3*^*-/-*^ mice, and then popliteal lymph node cells were fused with P3-X63.Ag8.653 mouse myeloma cells. Hybridomas producing anti-mouse E-NPP3 antibodies were cloned by limiting dilution. An anti-mouse E-NPP3 antibody purified by affinity chromatography using protein G-sepharose (GE Healthcare) was labeled with Alexa Fluor 647 using an Alexa Fluor 647 monoclonal antibody labeling kit (Invitrogen).

### Immunohistochemical staining

OCT compound (Leica)-embedded small intestine samples were sectioned and stained with the anti-mouse E-NPP3-Alexa Fluor 647 antibody. Images were obtained using an FV1000-D confocal microscope (Olympus).

### Measurement of ATP

ATP concentrations in the small intestinal lumen were measured as described previously [31]. In brief, the mice were fasted overnight and then anesthetized with sevoflurane (Abbvie, North Chicago, IL, USA). The peritoneal cavity was opened and the small intestine was ligated with silk threads at every 3 cm. A total of 300 μl PBS was injected into each loop. At 15 min after injection, luminal fluid was collected, and the concentration of ATP in the fluid was measured with an ATP assay kit (Toyo Ink, Tokyo, Japan).

### Mast cell reconstitution

To obtain bone marrow-derived mast cells, bone marrow cells from wild-type and *Enpp3*^*-/-*^ mice were cultured with 5 ng/ml recombinant murine IL-3 (Pepro Tech) for 3 weeks. CD3^-^, CD4^-^, CD8^-^, B220^-^, FcεRI^+^ and c-kit^+^ bone marrow-derived mast cells were purified using a FACS Aria (BD Biosciences), and 2×10^6^ mast cells were intravenously transferred into *Enpp3*^*-/-*^ *Kit*^*w-sh/w-sh*^ mice. At 2 and 6 days after the transfer, the number of mast cells in the small intestinal lamina propria was analyzed. Luminal ATP concentration was analyzed 6 days after the transfer.

### Cell isolation

Isolated PPs from the small intestine were incubated in HBSS (Nacalai Tesque, Kyoto, Japan) containing 5 mM EDTA at 37°C for 15 min to remove epithelial cells. After washing with cold PBS, PPs and MLNs were chopped finely and incubated in RPMI 1640 (Nacalai Tesque) containing 10% fetal bovine serum (FBS) and 0.5 mg/ml collagenase D (Roche, Indianapolis, IN, USA) at 37°C for 20 min and then pass through a 40-μm Cell Strainer (BD Biosciences) to obtain single-cell suspensions. Small intestinal lamina propria lymphocytes were isolated using a previously described protocol [14,31].

### Flow cytometry

Flow cytometric analysis was performed using a FACS Canto II flow cytometer (BD Biosciences) with FlowJo software (Tree Star, Ashland, OR, USA). pDCs and cDCs were isolated from MLNs, small intestinal lamina propria, PPs, bone marrow and spleens by using a FACS Aria (BD Biosciences). Instrumental settings were adjusted for four-color-stained samples.

### Characterization of pDCs

Surface markers of pDCs were analyzed by flow cytometery. SILP cells gated on CD45^+^ PDCA-1^+^ CD11c^med^ were analyzed for expression of Siglec H, CCR9 and CD45RA. For cytokine production analysis, CD45^+^ PDCA-1^+^ CD11c^med^ pDCs were isolated from SILP of wild-type and *Enpp3*^*-/-*^ mice by FACS Aria (BD Biosciences). The isolated pDCs were stimulated with or without CpG DNA (5μM) for 4 h. Expression of *Ifna1* and *Il12b* was analyzed by quantitative RT-PCR.

### Antibiotic treatment

Four-week-old mice were administrated combinations of four antibiotics including 1 mg/ml ampicillin (Nacalai Tesque), 1 mg/ml neomycin (Nacalai Tesque), 1 mg/ml metronidazole (Nacalai Tesque) and 500 μg/ml vancomycin (Duchefa Biochemie B.V.) in sterilized drinking water for 8 weeks.

### Apoptosis assay

Isolated cells from MLNs were cultured in RPMI 1640 medium containing 10% FBS, 100 U/ml penicillin (Gibco, Grand Island, NE, USA), 100 μg/ml streptomycin (Gibco), 50 μM 2-mercaptoethanol (Gibco) and 20 ng/ml recombinant murine IL-4 (PeproTech, Rocky Hill, NJ) at 37°C with or without ATP (Sigma). After 3 h, the cells were washed in PBS and stained with annexin V and an anti-active caspase-3 antibody as well as PDCA-1 and CD11c antibodies. The frequencies of annexin V- and active caspase-3-positive cells among CD11c^med^ PDCA-1^+^ pDCs and CD11c^high^ cDCs were analyzed by flow cytometry. In some experiments, CD45^+^ PDCA-1^+^ and CD11c^med^ cells were isolated from MLN cells by a FACS Aria (BD Biosciences) and treated with ATP for 3 h, and then stained with annexin V and an anti-active caspase-3 antibody.

### Induction of apoptosis *in vivo*

BALB/c mice were fasted overnight, anesthetized, and then the peritoneal cavity was opened. The proximal portion of the small intestine, which included PPs, was ligated with silk thread at every 3 cm. A total of 300 μl of 1 mM ATP-γS (Sigma) or PBS was injected into each loop, and then the peritoneal cavity was closed. After 4 h, PPs and small intestines were collected and cells were stained with anti-active caspase-3 antibody. The frequencies of active caspase-3-positive pDCs and cDCs in PPs were analyzed by flow cytometry.

### Statistical analysis

Comparisons between two groups were performed by Student’s t-test. Differences between multiple groups were analyzed using Tukey’s test followed by one-way ANOVA with JMP Pro 12 software. Differences with *P* < 0.05 were considered to be significant. Results are expressed as the mean ± SD.

## Results

### High expression of *Enpp3* in small intestinal epithelia

**Fig 1:**
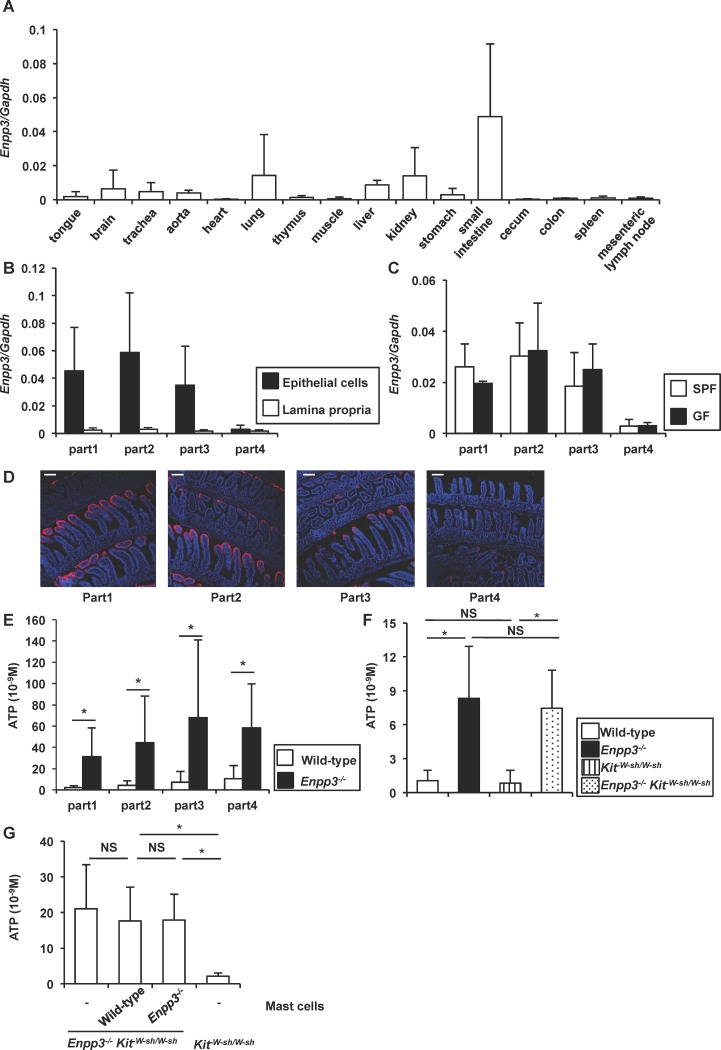
Expression of *Enpp3* in small intestinal epithelia. **(A)** Quantitative RT-PCR analysis of *Enpp3* mRNA expression in the indicated tissues (n = 3). **(B)** Quantitative RT-PCR analysis of *Enpp3* mRNA expression in epithelial cells and lamina propria in four parts of the small intestine. A smaller number represents a more proximal portion of the intestine (n = 4). **(C)** Quantitative RT-PCR analysis of *Enpp3* mRNA expression in epithelial cells of the small intestine in specific-pathogen free (SPF) and germ-free (GF) mice (n = 3). **(D)** Immunohistochemical analysis of the small intestine. E-NPP3 (red) and DAPI (blue). Swiss roll frozen sections were stained with the anti-mouse E-NPP3 antibody. A smaller number represents more proximal portion of the intestine. Scale bars, 100 μm. **(E)** ATP concentrations in luminal contents of the small intestine of wild-type and *Enpp3*^*-/-*^ mice. All data are mean values ± SD (n = 6 per groups). *p < 0.05. **(F)** ATP concentration in the proximal portion of the small intestinal lumen from wild-type, *Enpp3*^*-/-*^, *Kit*^*W-sh/W-sh*^ and *Enpp3*^*-/-*^ *Kit*^*W-sh/W-sh*^ mice. All data are mean values ± SD (n = 4 per groups). *p < 0.05, NS: not significant. **(G)** ATP concentration in the proximal portion of the small intestinal lumen from *Kit*^*W-sh/W-sh*^ and *Enpp3*^*-/-*^ *Kit*^*W-sh/W-sh*^ mice with or without adoptive transfer of wild-type or *Enpp3*^*-/-*^ bone marrow-derived mast cells. All data are mean values ± SD (n = 5 for mast cells transferred mice groups and *Kit*^*W-sh/W-sh*^ mice group, and n = 3 for non transferred *Enpp3*^*-/-*^ *Kit*^*W-sh/W-sh*^ mice group). *p < 0.05, NS: not significant.

*Enpp3* is highly expressed on epithelia of the human retina and endometrium as well as activated basophils and mast cells [10,39,40]. Therefore, we analyzed expression of *Enpp3* in mouse tissues by quantitative RT-PCR. *Enpp3* was highly expressed in the small intestine (Fig 1A). In the small intestine, *Enpp3* was expressed at a higher level in the epithelial layer than in the lamina propria (Fig 1B). *Enpp3* expression was highly observed in the proximal portion of the small intestinal epithelia (Fig 1B). *Enpp3* expression was not reduced in germ-free mice, in which intestinal microbiota was absent, suggesting that *Enpp3* expression is not dependent on intestinal bacteria (Fig 1C). We also analyzed protein expression of E-NPP3 in the small intestine by immunohistochemistry (Fig 1D). Similar to the *Enpp3* mRNA expression pattern, protein expression of E-NPP3 was higher in the proximal portion than that in the distal portion of the small intestine. In addition, E-NPP3 was expressed in the epithelial layer at the tip of villi in the small intestine. Therefore, we determined whether the ATP-hydrolyzing ectoenzyme E-NPP3 regulates the ATP concentration in the small intestinal lumen using *Enpp3*^*-/-*^ mice. Ligated intestinal loops of four parts from proximal to distal regions of the small intestine were created, and then PBS was injected into the loop. After 15 min, the concentration of ATP was analyzed in the luminal content. ATP concentrations in the lumens of all parts were elevated in the small intestine of *Enpp3*^*-/-*^ mice (Fig 1E). Because E-NPP3 is highly expressed in activated mast cells, which are abundant in the intestinal lamina propria [10], we investigated whether E-NPP3 on mast cells contributes to controlling luminal ATP by crossing *Enpp3*^*-/-*^ mice with *Kit*^*W-sh/W-sh*^ mice that lack mast cells. In *Enpp3*^*-/-*^ *Kit*^*W-sh/W-sh*^ mice, the luminal ATP concentration in the small intestine was increased to a similar level as that in *Enpp3*^*-/-*^ mice (Fig 1F). We then transferred wild-type or *Enpp3*^*-/-*^ bone marrow-derived mast cells into *Enpp3*^*-/-*^ *Kit*^*W-sh/W-sh*^ mice, and analyzed luminal ATP concentration (Fig 1G). Transferred bone marrow-derived mast cells were present in the small intestinal lamina propria at 2 days after the transfer (S1 Fig). Adoptive transfer of wild-type mast cells did not decrease the luminal ATP concentration in *Enpp3*^*-/-*^ *Kit*^*W-sh/W-sh*^ mice at 6 days after the transfer, demonstrating that E-NPP3 on mast cells does not control the ATP concentration in the intestinal lumen. These findings indicate that E-NPP3 is highly expressed on the epithelial layer of the small intestine and controls the luminal ATP concentration.

### Reduced number of pDCs in *Enpp3*^*-/-*^ intestines

**Fig 2:**
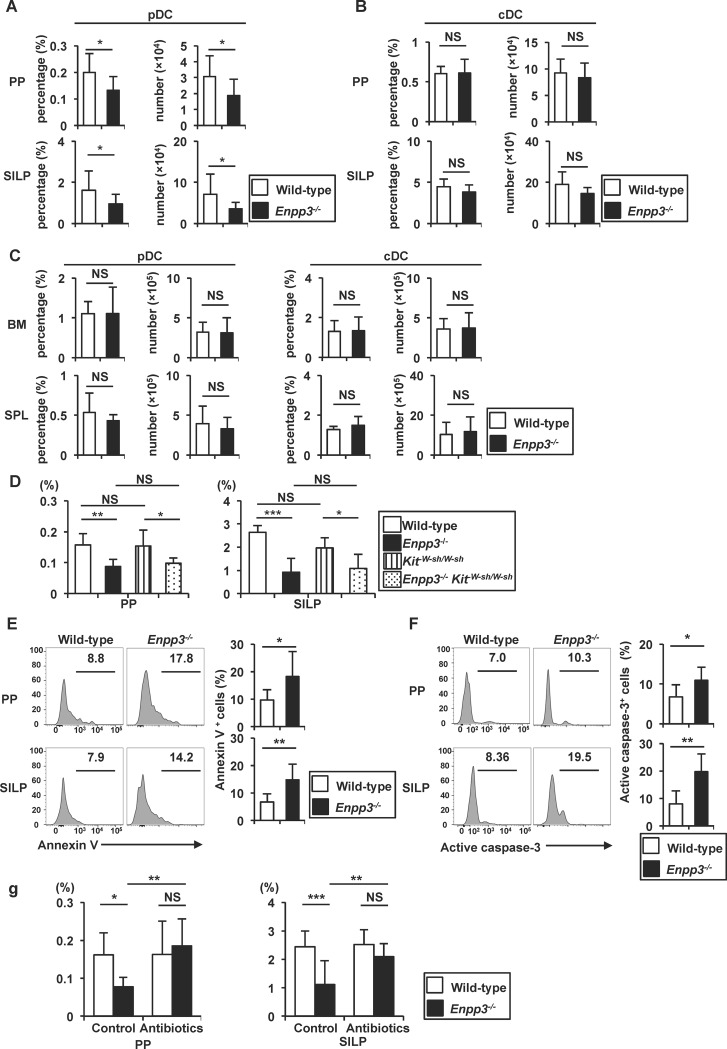
Decrease in the number of intestinal pDCs in *Enpp3*^*-/-*^ mice. **(A**–**C)** Frequency and number of CD45^+^ PDCA-1^+^ CD11c^med^ pDCs (A, C) and CD45^+^ PDCA-1^-^ CD11c ^high^ cDCs (B, C) in the Peyer’s patches (PPs), small intestinal lamina propria (SILP), bone marrow (BM), and spleen (SPL) of wild-type and *Enpp3*^*-/-*^ mice. All data are mean values ± SD (n = 7 for PP and SILP, and n = 6 for SPL and BM). *p < 0.05, NS: not significant. **(D)** Frequency of CD45^+^ PDCA-1^+^ CD11c^med^ pDCs in the PPs and SILP of wild-type (n = 6), *Enpp3*^*-/-*^ (n = 8), *Kit*^*W-sh/W-sh*^ (n = 7), and *Enpp3*^*-/-*^ *Kit*^*W-sh/W-sh*^ (n = 6) mice. All data are mean values ± SD. *p < 0.05, **p < 0.01, ***p < 0.001, NS: not significant. **(E, F)** Frequency of annexin V-positive (E) and active caspase-3-positive (F) cells gated on CD45^+^ PDCA-1^+^ CD11c^med^ pDCs from the PPs and SILP of wild-type and *Enpp3*^*-/-*^ mice. Representative histograms are shown (left) and the means ± SD of the percentages of positive cells (right) are shown (n = 6 in e, and n = 5 in f). *p < 0.05, **p < 0.01. **(G)** Frequency of PDCA-1^+^ CD11c^med^ pDCs in the PPs and SILP from antibiotic-treated wild-type (n = 11) and *Enpp3*^*-/-*^ (n = 12) mice as well as untreated wild-type (n = 10) and *Enpp3*^*-/-*^ (n = 10) mice. Data are the means ± SD of the percentages of pDCs. *p < 0.05, **p < 0.01, ***p < 0.001, NS: not significant.

Luminal ATP induces differentiation of IL-17-producing T cells through activation of a unique subtype of lamia propria DCs [14]. Furthermore, *Entpd7*^*-/-*^ mice show increased numbers of IL-17-producing T cells in the small intestinal lamia propria (SILP) with an elevated luminal ATP concentration [31]. Therefore, we analyzed the number of IL-17-producing T cells and other immune cells in the intestines of *Enpp3*^*-/-*^ mice. The numbers of B220^+^ cells and CD4^+^ cells were not altered in the SILP and PPs of *Enpp3*^*-/-*^ mice (S2A Fig). However, the number of IL-17-producing T cells was slightly increased in the SILP of *Enpp3*^*-/-*^ mice (S2B Fig). Although ATP has been shown to control follicular T helper (Tfh) cell numbers in PPs [21], the frequency and number of Tfh cells was not altered in PPs of *Enpp3*^*-/-*^ mice (S2C Fig). We also analyzed other immune cell populations, such as pDCs and conventional DCs (cDCs). The frequency and number of CD11c^med^ PDCA-1^+^ pDCs, but not CD11c^high^ cDCs, were decreased in the SILP and PPs of *Enpp3*^*-/-*^ mice compared with wild-type mice (Fig 2A and 2B and S3A Fig). We next analyzed the numbers of pDCs and cDCs in the bone marrow and spleen (Fig 2C and S3B Fig). The frequency and number of CD11c^med^ PDCA-1^+^ pDCs and CD11c^high^ cDCs were not altered in the bone marrow and spleen of *Enpp3*^*-/-*^ mice. The number of pDCs in the SILP and PPs was also reduced in *Enpp3*^*-/-*^ *Kit*^*W-sh/W-sh*^ mice (Fig 2D). Thus, the number of pDCs was decreased selectively in the intestines of *Enpp3*^*-/-*^ mice.

We next analyzed the function of intestinal pDCs of *Enpp3*^*-/-*^ mice. Surface expression of pDC markers in pDCs in SILP was first analyzed by flow cytometry (S4A Fig). Expression of Siglec H, which is selectively expressed in pDCs, was not altered in *Enpp3*^*-/-*^ pDCs. CCR9, which was highly expressed in pDC migrating into the small intestine, was also normally expressed in *Enpp3*^*-/-*^ pDCs. CD45RA, which was expressed higher in pDCs than cDCs, was not reduced in *Enpp3*^*-/-*^ pDCs. We then analyzed cytokine production from SILP pDCs. pDCs were isolated from SILP of wild type and *Enpp3*^*-/-*^ mice, stimulated with CpG DNA, and analyzed for expression of *Ifna1* (encoding IFN-α) and *Il12b* (encoding IL-12p40) by quantitative RT-PCR (S4B Fig). *Enpp3*^*-/-*^ pDCs showed normal expression of these cytokines. Thus, the functions of pDCs in the small intestine of *Enpp3*^*-/-*^ mice were not impaired.

We next analyzed whether intestinal pDCs of *Enpp3*^*-/-*^ mice were sensitive to cell death by staining with annexin V (Fig 2E). Annexin V-positive pDCs was increased in PPs and SILP of *Enpp3*^*-/-*^ mice compared with wild-type mice. We also stained pDCs with an antibody that detects active caspase-3 produced during the process of apoptosis (Fig 2F). There were increased numbers of pDCs positive for active caspase-3 in PPs and SILP of *Enpp3*^*-/-*^ mice compared with wild-type mice. These findings indicate a decrease of intestinal pDCs due to the enhanced apoptosis in *Enpp3*^*-/-*^ mice. Because the intestinal ATP concentration increases in a commensal microbiota-dependent manner [14], we examined whether commensal microbiota contribute to the reduction of intestinal pDC numbers (Fig 2G and S3C Fig). Wild-type and *Enpp3*^*-/-*^ mice were orally treated with combinations of four antibiotics (vancomycin, streptomycin, metronidazole, and ampicillin) for 8 weeks. In antibiotic-treated *Enpp3*^*-/-*^ mice, the numbers of pDCs in the SILP and PPs were increased compared with those in untreated *Enpp3*^*-/-*^ mice. Taken together, these findings indicate that intestinal pDCs decrease because of the increase in the concentration of intestinal ATP, which was dependent on commensal microbiota in the absence of E-NPP3.

### Induction of pDC apoptosis by extracellular ATP

**Fig 3:**
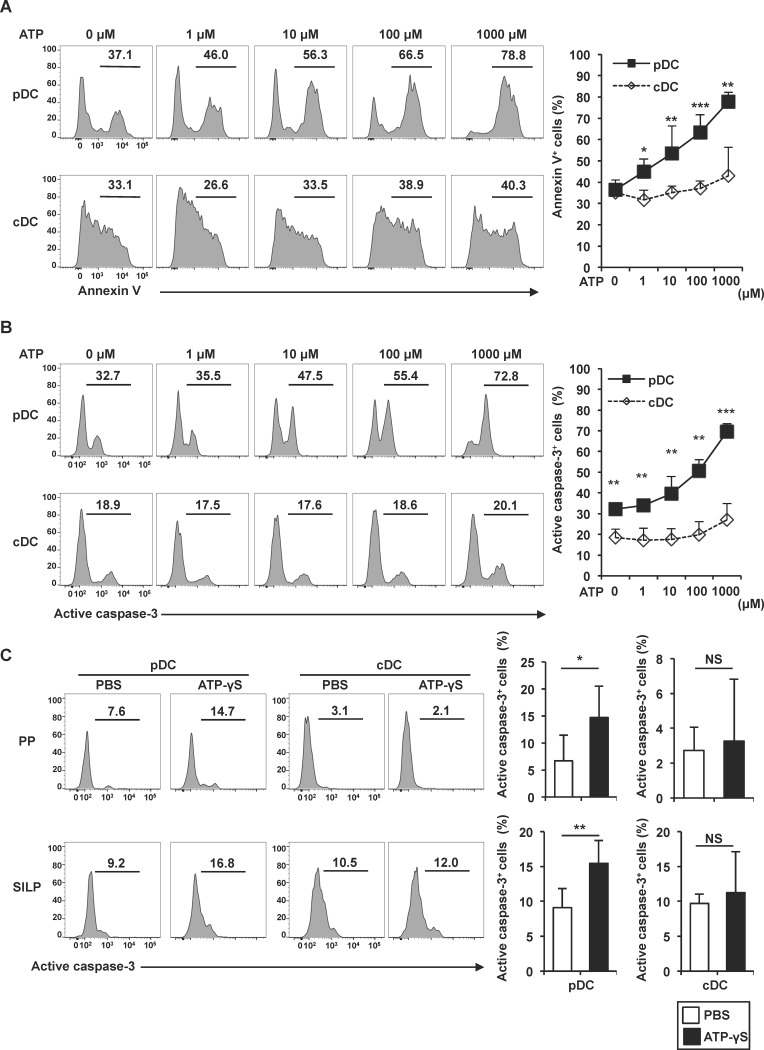
Enhancement of ATP-induced apoptosis of pDCs. **(A, B)** Cells isolated from MLNs were treated with the indicated concentrations of ATP for 3 h. Annexin V-positive cells (A) and active caspase-3-positive cells (B) among PDCA-1^+^ CD11c^med^ pDCs and PDCA-1^-^ CD11c^high^ cDCs were analyzed by flow cytometry. Representative histograms are shown (left) and the means ± SD (n = 3) of the percentages of annexin V-positive (A) and active caspase-3-positive cells (B) are shown (right). *p < 0.05, **p < 0.01, ***p < 0.001. **(C)** Frequency of active caspase-3-positive cells among CD45^+^ PDCA-1^+^ CD11c^med^ pDCs and and PDCA-1^-^ CD11c^high^ cDCs from the PPs and SILP of mice injected with 300 μl PBS (n = 6) or 1 mM ATP-γS (n = 5) into their small intestinal lumen. Representative histograms are shown (left) and the means ± SD of the percentages of positive cells are shown (right). *p < 0.05, **p < 0.01, NS: not significant.

We next analyzed the mechanisms responsible for the reduced number of pDCs in the intestine. Several studies have indicated that extracellular ATP induces cell death in many cell types including monocytes/macrophages, DCs, and T cells [15,18,19,21,41]. Therefore, we determined whether extracellular ATP induces cell death of pDCs. MLN cells were isolated, treated with increasing concentrations of ATP for 3 h, and analyzed for annexin V binding by flow cytometry in pDC and cDC populations (Fig 3A). ATP did not increase annexin V-positive cDCs, but increased annexin V binding to pDCs in a dose-dependent manner. Then, the cells were analyzed for expression of active caspase-3 (Fig 3B). ATP treatment showed dose-dependent enhancement of active caspase-3 in pDCs, but not in cDCs. These results indicate that pDCs are highly sensitive to ATP-induced cell death. We next analyzed the *in vivo* effect of ATP on apoptosis of intestinal pDCs. Non-hydrolyzable ATP, ATP-γS, was administered directly into the small intestine. At 4 h after administration, pDCs and cDCs in PPs and SILP were analyzed for expression of active caspase-3 (Fig 3C). Luminal administration of ATP-γS increased active caspase-3 in pDCs, but not in cDCs. Thus, ATP induced cell death of intestinal pDCs *in vitro* and *in vivo*.

### P2X7 mediates ATP-induced pDC cell death

**Fig 4:**
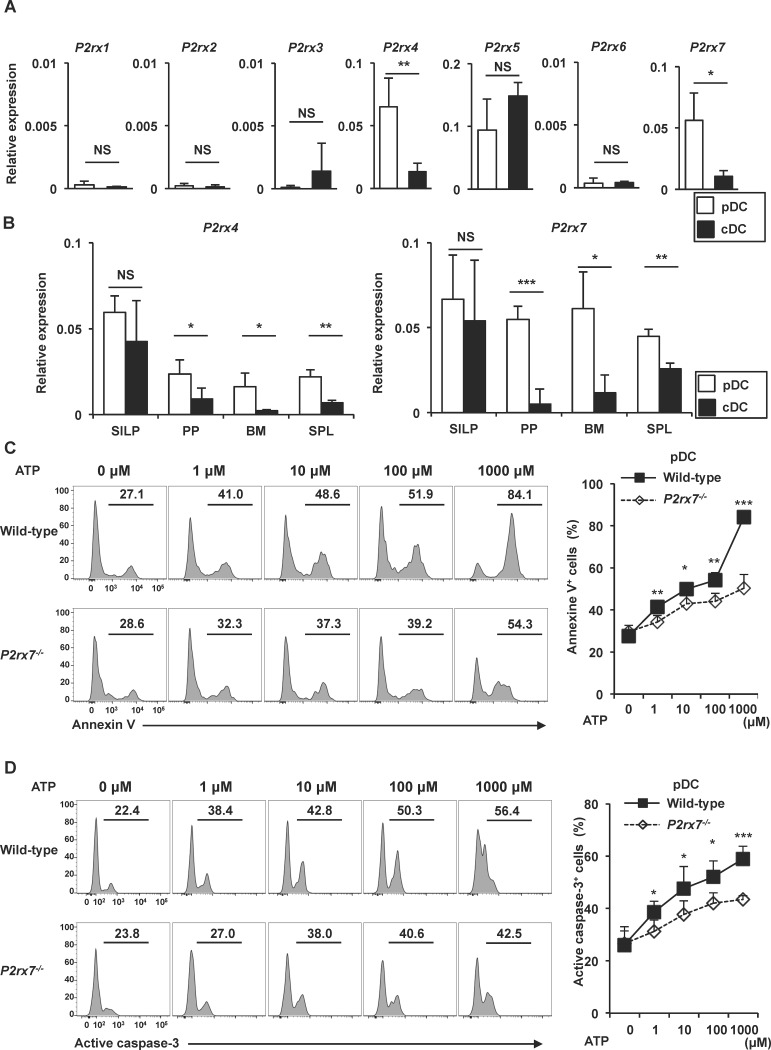
P2X7-dependent induction of pDC apoptosis in vitro. **(A)** Expression of P2X receptors in pDCs or cDCs of MLN was analyzed by quantitative RT-PCR. Data are means ± SD (n = 3). *p < 0.05, **p < 0.01. **(B)** mRNA expression of *P2rx4* and *P2rx7* in pDCs and cDCs of SILP, PP, bone marrow (BM) and spleen (SPL) was analyzed by quantitative RT-PCR. Data are means ± SD (n = 3). *p < 0.05, **p < 0.01. **(C, D)** Isolated MLN pDCs of wild-type and *P2rx7*^*-/-*^ mice were treated with the indicated concentrations of ATP for 3 h. Annexin V-positive cells (C) and active caspase-3-positive cells (D) were analyzed by flow cytometry. Representative histograms are shown (left) and the means ± SD of the percentages of annexin V-positive (C) and active caspase-3-positive cells (D) (n = 4) are shown (right). *p < 0.05, **p < 0.01, ***p < 0.001.

We next analyzed the mechanisms by which intestinal pDCs are highly sensitive to ATP-induced cell death. The ATP sensors, P2X4 and P2X7, are involved in ATP-induced cell death of many cell types [19,41–43]. Therefore, we analyzed mRNA expression of P2X receptors in pDCs and cDCs of MLN by quantitative RT-PCR (Fig 4A). pDCs expressed higher level of *P2rx4* and *P2rx7* than cDCs. We next compared expression levels of *P2rx4* and *P2rx7* in pDCs and cDCs of several tissues, such as SILP, PP, bone marrow, and spleen (Fig 4B). In accordance with the fact that ATP activates intestinal cDCs [14], both pDCs and cDCs in SILP similarly and highly expressed *P2rx4* and *P2rx7*. In all the tissues analyzed (SILP, PP, MLN, bone marrow and spleen), high expression of *P2rx7* in pDCs, but not cDCs, was observed. In contrast, expression level of *P2rx4* in pDCs was not so high in PP, bone marrow, and spleen, although it was expressed at high level in MLN and SILP. Therefore, we focused on P2X7, and we examined ATP-induced cell death of pDCs in *P2rx7*^*-/-*^ mice. pDCs were isolated from MLN of wild-type and *P2rx7*^*-/-*^ mice, treated with the indicated concentrations of ATP for 3 h, and then analyzed for annexin V binding and active caspase-3 in the pDC population (Fig 4C and 4D). ATP treatment enhanced annexin V binding and active caspase-3 in wild-type pDCs in a dose-dependent manner. However, the ATP-dependent increased of annexin V binding- and active caspase-3-positive cells were reduced in *P2rx7*^*-/-*^ pDCs compared to those in wild-type cells. These findings indicate that ATP induces cell death of pDCs via P2X7.

**Fig 5:**
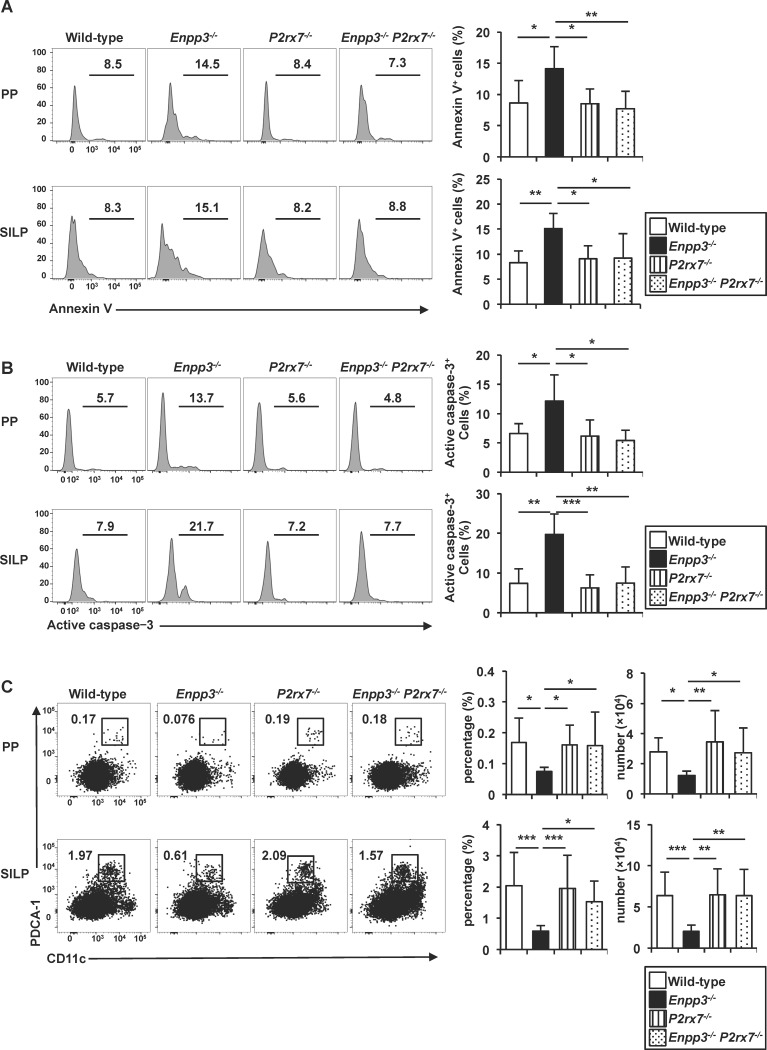
P2X7-dependent induction of pDC apoptosis in vivo. **(A, B)** Frequencies of annexin V-positive (A) and active caspase-3-positive (B) cells gated on CD45^+^ PDCA-1^+^ CD11c^med^ pDCs from the PPs and SILP of wild-type, *Enpp3*^*-/-*^, *P2rx7*^*-/-*^, and *Enpp3*^*-/-*^ *P2rx7*^*-/-*^ mice (n = 7 per groups in a, and n = 5 per groups in b). Representative histograms are shown (left) and the means ± SD of the percentages of annexin V-positive (A) and active caspase-3-positive cells (B) are shown (right). *p < 0.05, **p < 0.01 **(c)** Frequency and number of PDCA-1^+^ CD11c^med^ pDCs in the PPs and SILP from wild-type, *Enpp3*^*-/-*^, *P2rx7*^*-/-*^, and *Enpp3*^*-/-*^ *P2rx7*^*-/-*^ (n = 13 per groups) mice. Representative dot plots are shown (left) and the means ± SD of the percentages of pDCs are shown (right). *p < 0.05, **p < 0.01, ***p < 0.001.

We next analyzed the *in vivo* role of P2X7 in the ATP-induced cell death of pDCs by inducing *P2rx7* deficiency in *Enpp3*^-/-^ mice. In *Enpp3*^-/-^ *P2rx7*^-/-^ mice, the numbers of annexin V- and active caspase-3-positive pDCs in PPs and SILP were dramatically reduced compared with those in *Enpp3*^-/-^ mice (Fig 5A and 5B). Accordingly, the numbers of pDCs in the PPs and SILP of *Enpp3*^-/-^ *P2rx7*^-/-^ mice were increased to comparable levels to those in wild-type mice (Fig 5C). Taken together, these findings suggest that P2X7 mediates the ATP-dependent cell death of intestinal pDCs.

## Discussion

E-NPP3 (also known as CD203c) regulates basophil- and mast cell-mediated chronic allergic inflammation [10]. In the present study, we demonstrate that E-NPP3 is highly expressed on small intestinal epithelial cells and regulates the luminal ATP concentration. In *Enpp3*^-/-^ mice, the number of intestinal pDCs was decreased, which was accompanied by an increased concentration of luminal ATP. Intestinal pDCs were highly sensitive to ATP-induced cell death. Thus, E-NPP3 is responsible for the maintenance of pDCs via the control of ATP concentrations in the small intestine.

Extracellular ATP induces immune responses by acting on different immune cell types via P2 receptors [1,2,9,44]. Conversely, several lines of evidence indicate that ATP induces cell death of several immune cell types to modulate immune responses [15–21]. The present study demonstrates that intestinal pDCs are highly sensitive to ATP-induced cell death. In the absence of P2X7, ATP-induced pDC cell death was severely inhibited. However, high concentration of ATP still induced pDC death of *P2rx7*^-/-^ mice. In this regard, *P2rx4*, which was also highly expressed in pDCs, might contribute to the ATP-induced cell death. *P2rx7* was also highly expressed in pDCs in other tissues such as spleen and bone marrow. Thus, P2X7 can be used as a general marker for pDCs. In addition, in a situation when extracellular ATP increases in those tissues, pDCs might decrease in number. Another intriguing point is that *P2rx4* and *P2rx7* were similarly expressed in pDCs and cDCs in SILP. Thus, cDCs in SILP were supposed to respond to ATP via P2X4 and P2X7, but showed no ATP-induced apoptotic response. In this regard, a subset of cDCs in the intestinal lamina propria showed ATP-induced gene expression [14]. Thus, pDCs and cDCs in SILP might possess differential properties in terms of ATP-induced responses, apoptosis and gene expression, respectively. It would be an interesting future issue to analyze ATP-induced activation of signaling pathways in pDCs and cDCs in SILP.

It is well known that pDCs play essential roles in host defense against viral infection through high production of type I IFNs [32,45]. Accordingly, chronic viral infection has been shown to cause decreases in the pDC number and type I IFN production in mice [46]. A decreased number of pDCs has also been found in patients with chronic infection by hepatitis B or C viruses [47,48]. Cell death of pDCs during viral infection has been shown to be mediated by type I IFN [49]. Thus, pDC numbers are tightly regulated during viral infections. The present study demonstrates that the numbers of intestinal pDCs are controlled by extracellular ATP. In the case of intestinal pDCs, cell death was directly induced by ATP, and did not appear to be dependent on autocrine type I IFNs based on the following two findings. First, an alteration in the number of pDCs in gut-associated lymphoid tissue has not been described in *Ifnar1*^-/-^ mice that lack type I IFN signaling [38]. Second, our unpublished results showed that mRNA levels of genes encoding type IFNs (*Ifna* and *Ifnb*) were not elevated in PPs or small intestinal tissues of *Enpp3*^-/-^ mice.

In addition to the host defense against viral infections, pDCs have been implicated in regulation of several immune responses, including T cell-independent induction of IgA-producing plasma cells and induction of oral tolerance [38] [50]. Accordingly, the number of IgA-expressing plasma cells in SILP was reduced in *Enpp3*^-/-^ mice (our unpublished results). Induction of oral tolerance was also partially reduced in *Enpp3*^-/-^ mice (our unpublished results). Although it remains unknown whether these impairments were due to the reduced number of intestinal pDCs, the deficiency of *Enpp3* leads to defective intestinal immune responses.

Considering that the intestinal mucosa is one of main routes for viral invasion, the regulation of pDCs by ATP is supposed to be associated with the host–virus interaction in the intestines. Several lines of evidence indicate that increased ATP production by host cells enhances viral growth [51,52]. Extracellular ATP also mediates infection and budding of human immunodeficiency virus 1 [53,54]. Thus, ATP in the extracellular and intracellular compartments is used by viruses for their propagation in the host. In addition, ATP increases cell death of intestinal pDCs, a major player in anti-viral responses. Therefore, viruses might promote ATP production by host cells for evasion of anti-viral immune responses induced by pDCs as well as promotion of viral growth in the intestines. The host, in turn, expresses E-NPP3 at the mucosal surface to decrease the concentration of ATP that might be harmful when produced in excess.

Tfh cells in PPs have been shown to be sensitive to P2X7-mediated cell death [21]. However, in the present study, the number of Tfh cells in PPs was not decreased in *Enpp3*^-/-^ mice. In this regard, it might be possible that both pDCs and Tfh cells are sensitive to ATP, but pDCs exhibit higher sensitivity to ATP than Tfh cells. Indeed, Tfh cells become resistant to ATP-mediated cell death by TCR stimulation [21]. Furthermore, a high concentration of ATP (over 1 mM) is required to induce Tfh cell death [21], whereas the micromolar levels of ATP induced cell death of intestinal pDCs.

Nucleotide-converting ectoenzymes, such as E-NTPD1, E-NTPD7, and E-NPP3 regulate ATP-mediated immune and inflammatory responses via ATP hydrolysis [10,31,55–57]. Thus, many studies have focused on negative regulation of immune responses through control of the extracellular ATP concentration. The present study demonstrates another aspect of the importance of the control of ATP that induces cell death and thus promotes disadvantageous conditions in terms of the maintenance of intestinal homeostasis. E-NPP3 expressed on the small intestinal epithelial cell layer controls the intestinal immune response via the prevention of pDC death in the intestines.

In the present study, we revealed that the ectoenzyme E-NPP3 regulates the number of small intestinal pDCs, which are involved in immune responses in the intestine. However, Th17 cell numbers in the small intestine also increased in *Enpp3*^-/-^ mice. A previous study has demonstrated that another ectoenzyme, E-NTPD7, which was also highly expressed on the epithelial cells of the small intestine, regulates Th17 cell responses [31]. It is possible that other ATP-hydrolyzing ectoenzymes are expressed on intestinal epithelial cell layers. Therefore, it would be an interesting future study to analyze the role of these ectoenzymes in the control of pDC numbers in the intestines. Elucidation of the role of the family of ATP-hydrolyzing ectoenzymes in the regulation of immune responses might lead to development of a novel strategy to modulate intestinal inflammation, such as inflammatory bowel diseases.

## Supporting information

S1 Fig Reconstitution of mast cells. Bone marrow-derived mast cells were transferred into *Kit*^*W-sh/W-sh*^ mice. At day 2 and day 6 after the reconstitution, CD3^-^ CD4^-^ CD8^-^ B220^-^ cells were gated and frequencies of mast cells in the small intestine were analyzed for expression of c-kit^+^ FcεRI^+^ by flow cytometory.
(TIFF)

S2 Fig Development of lymphocytes in the intestine of *Enpp3*^*-/-*^ mice. (A) Frequency and number of CD4^+^ T cells and B220^+^ B cells in the PPs and SILP of wild-type (n = 6) and *Enpp3*^*-/-*^ (n = 6) mice. Representative dot plots are shown (left) and the means ± SD of the percentages and total numbers of CD4^+^ or B220^+^ cells are shown (right). NS: not significant. (B) Frequency and number of IL-17-producing CD4^+^ T cells in the small intestine of wild-type (n = 5) and *Enpp3*^*-/-*^ (n = 5) mice. Representative dot plots are shown (left) and the means ± SD of the percentages and total numbers of IL-17^+^ CD4^+^ cells are shown (right). *p < 0.05, NS: not significant. (C) Frequency and numbers of CD4^+^ ICOS^+^ CXCR5^+^ follicular helper T (Tfh) cells in the PPs of wild-type (n = 5) and *Enpp3*^*-/-*^ (n = 5) mice. Representative dot plots are shown (left) and the means ± SD of the percentages and total numbers of Tfh cells are shown (right). NS: not significant.
(TIFF)

S3 Fig Decrease in the number of intestinal pDCs in *Enpp3*^*-/-*^ mice. (A, B) Frequency of CD45^+^ PDCA-1^+^ CD11c^int^ pDCs and CD45^+^ PDCA-1^-^ CD11c ^high^ cDCs in the PPs, SILP (A), BM, and SPL (B) of wild-type and *Enpp3*^*-/-*^ mice. Representative dot plots are shown. Numbers in dot plots indicate the percentages of cells in the respective areas. (C) Frequency of PDCA-1^+^ CD11c^int^ pDCs in the PPs and SILP from antibiotic-treated wild-type (n = 11) and *Enpp3*^*-/-*^ (n = 12) mice or untreated wild-type (n = 10) and *Enpp3*^*-/-*^ (n = 10) mice. Representative dot plots are shown. Numbers in dot plots indicate the percentages of cells in the respective areas.
(TIFF)

S4 Fig The function of intestinal pDCs of *Enpp3*^*-/-*^ mice. (A) Surface expression of Siglec H, CCR9 and CD45RA on CD45^+^ PDCA-1^+^ CD11c^med^ pDCs from SILP analyzed by flow cytometry. (B) CD45^+^ PDCA-1^+^ CD11c^med^ pDCs were isolated from SILP of wild-type and *Enpp3*^*-/-*^ mice with FACS Aria. pDCs were stimulated with CpG DNA (5 μM) for 4 h. Expression of *Ifna1* and *Il12b* was analyzed by quantitative RT-PCR (n = 3). NS: not significant.
(TIFF)
