# Supplementary figures and images for: E-NPP3 controls plasmacytoid dendritic cell numbers in the small intestine
Source: PLoS One. 2017 Feb 22;12(2):e0172509. doi: 10.1371/journal.pone.0172509 (PMC5321438; doi:10.1371/journal.pone.0172509)

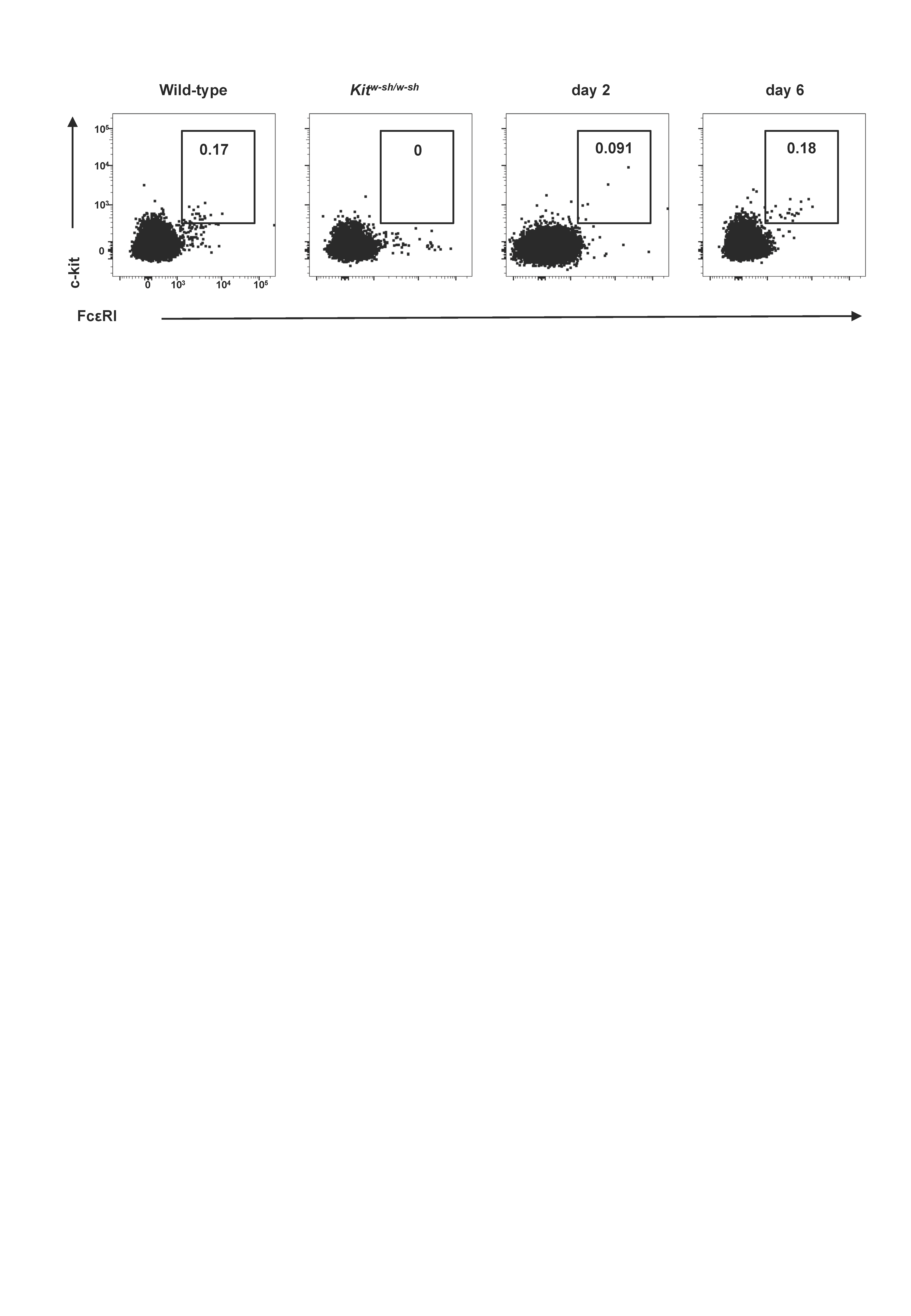

Supplement: S1 Fig — Bone marrow-derived mast cells were transferred into KitW-sh/W-sh mice. At day 2 and day 6 after the reconstitution, CD3- CD4- CD8- B220- cells were gated and frequencies of mast cells in the small intestine were analyzed for expression of c-kit+ FcεRI+ by flow cytometory. (TIFF) [file pone.0172509.s001.tiff]

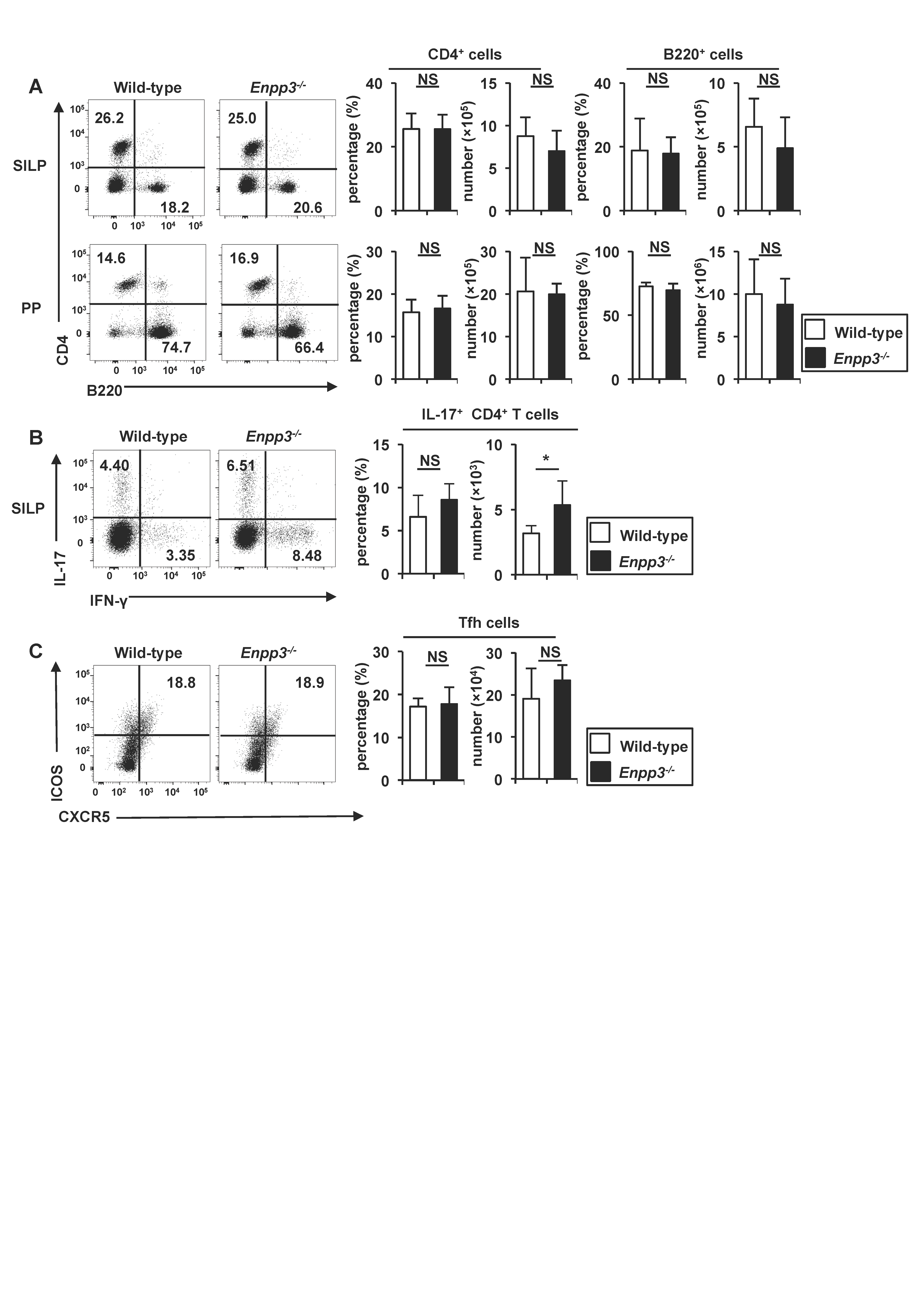

Supplement: S2 Fig — (A) Frequency and number of CD4+ T cells and B220+ B cells in the PPs and SILP of wild-type (n = 6) and Enpp3-/- (n = 6) mice. Representative dot plots are shown (left) and the means ± SD of the percentages and total numbers of CD4+ or B220+ cells are shown (right). NS: not significant. (B) Frequency and number of IL-17-producing CD4+ T cells in the small intestine of wild-type (n = 5) and Enpp3-/- (n = 5) mice. Representative dot plots are shown (left) and the means ± SD of the percentages and total numbers of IL-17+ CD4+ cells are shown (right). *p < 0.05, NS: not significant. (C) Frequency and numbers of CD4+ ICOS+ CXCR5+ follicular helper T (Tfh) cells in the PPs of wild-type (n = 5) and Enpp3-/- (n = 5) mice. Representative dot plots are shown (left) and the means ± SD of the percentages and total numbers of Tfh cells are shown (right). NS: not significant. (TIFF) [file pone.0172509.s002.tiff]

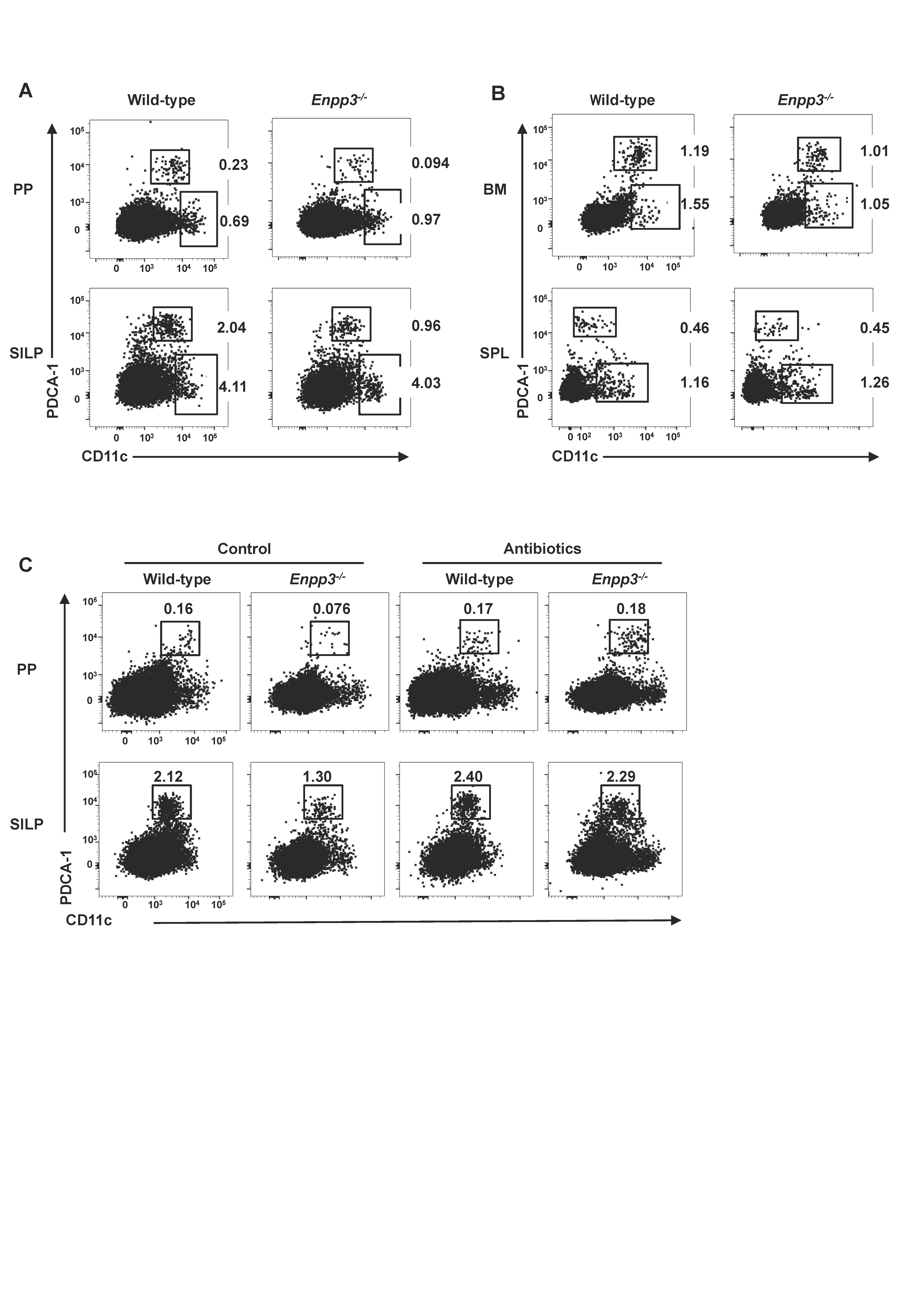

Supplement: S3 Fig — (A, B) Frequency of CD45+ PDCA-1+ CD11cint pDCs and CD45+ PDCA-1- CD11c high cDCs in the PPs, SILP (A), BM, and SPL (B) of wild-type and Enpp3-/- mice. Representative dot plots are shown. Numbers in dot plots indicate the percentages of cells in the respective areas. (C) Frequency of PDCA-1+ CD11cint pDCs in the PPs and SILP from antibiotic-treated wild-type (n = 11) and Enpp3-/- (n = 12) mice or untreated wild-type (n = 10) and Enpp3-/- (n = 10) mice. Representative dot plots are shown. Numbers in dot plots indicate the percentages of cells in the respective areas. (TIFF) [file pone.0172509.s003.tiff]

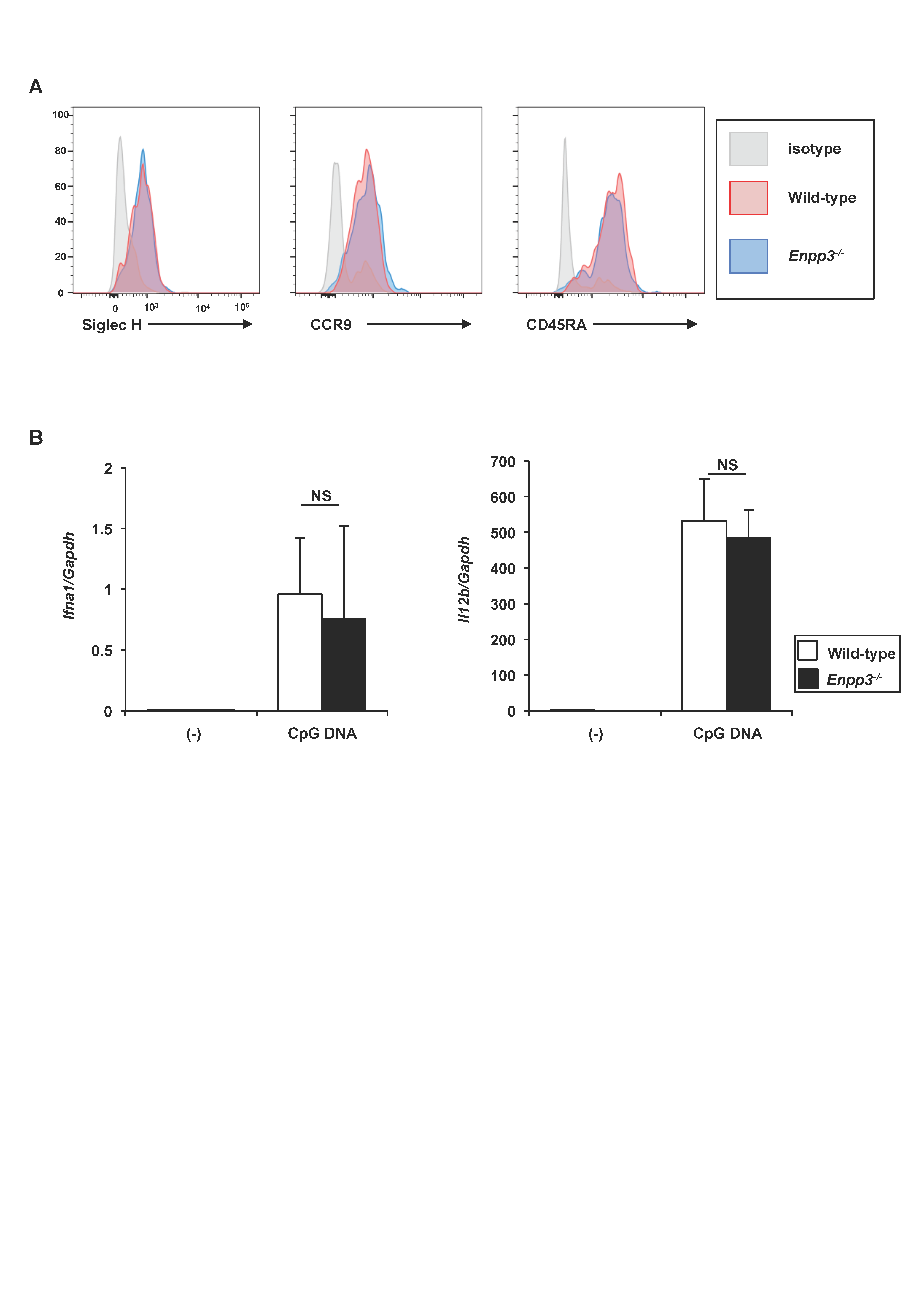

Supplement: S4 Fig — (A) Surface expression of Siglec H, CCR9 and CD45RA on CD45+ PDCA-1+ CD11cmed pDCs from SILP analyzed by flow cytometry. (B) CD45+ PDCA-1+ CD11cmed pDCs were isolated from SILP of wild-type and Enpp3-/- mice with FACS Aria. pDCs were stimulated with CpG DNA (5 μM) for 4 h. Expression of Ifna1 and Il12b was analyzed by quantitative RT-PCR (n = 3). NS: not significant. (TIFF) [file pone.0172509.s004.tiff]
